# A Treatise on Oral Deformities as a Branch of Mechanical Surgery

**Published:** 1880-04

**Authors:** 


### BIBLIOGRAPHICAL.

---

*A Treatise on Oral Deformities as a Branch of Mechanical Surgery.* By Norman W. Kingsley, D. D. S. Publishers: D. Appleton & Co., New York, 1880.

The author is well known to the dental profession as an authority on such subjects as are treated of in this work, which consists of a handsome volume of nearly six hundred pages, illustrated by over three hundred and fifty engravings.

The subject of "Irregularity of the Teeth" is first noticed, with a full and comprehensive explanation of how such conditions may be remedied and the appliances necessary to be constructed. The article on "Palatine Defects" relating to cleft palate, and hare-lip, is an admirable one and contains much that is useful to the practitioner. "Mechanism of Speech" and "The Æsthetics of Dentistry" reveal the art-knowledge of the author, and the necessity existing for the dental practitioner to become informed concerning the established results of the many experiments made by the author of this treatise. The importance of restoring the natural *expression* is recognized by everyone concerned in the manufacture of artificial substitutes, and requires a proper knowledge and use of the laws which define the difference between an art and mere mechanical skill.

Sir Charles Bell's, and the late Prof. McQuillen's views are adopted as authority, and the author is governed by the laws found in the former's "Anatomy of Expression" for his practice, and for securing the artistic realization of the harmony of the features. This new work is well worthy of its author and furnishes an important addition to the literature of the dental profession.

